# Preliminary Evaluation of a Tris-Based Extender for the Semen Examination Procedure in a Hybrid Falcon (*Falco rusticolus × Falco cherrug*)

**DOI:** 10.1155/crve/6233188

**Published:** 2025-10-01

**Authors:** Miranda Govoni, Chiara Milani, Marco Martini, Barbara Contiero, Gabriele Gerardi, Antonio Mollo, Severino Segato

**Affiliations:** ^1^Department of Animal Medicine, Production and Health, Padova University, Padova, Italy; ^2^Urbeveteris Falconis Center, Terni, Italy

**Keywords:** Gyr-saker hybrid falcon, semen, sperm motility, sperm viability, Tris-based extender

## Abstract

Artificial insemination (AI) plays an important role in threatened raptor species conservation, as well as in falconry for commercial purposes. There is still a need to identify a suitable extender that maintains an adequate environment for sperm function. The present report was aimed at describing the proficient use of a Tris-based extender (TbE) applied during AI procedure in a hybrid falcon (*Falco rusticolus* × *Falco cherrug*). During the reproductive season, a total of 15 semen samples of a hand-reared, 11-year-old Gyr-saker hybrid falcon were analyzed for volume, color, pH, and concentration. Sperm viability was tested on undiluted and TbE-diluted samples, while total and progressive motility was assessed according to the use of extender after 0 (T0), 20 (T20), or 40 (T40) minutes from semen collection. The dilution with TbE did not result in any significant difference in motility parameters compared to undiluted samples and did not cause significant alteration in sperm viability. TbE was able to support sperm motility for at least 40 min at room temperature.

## 1. Introduction

Artificial insemination (AI) in avian species plays an important role, especially related to the increase of production and advancement of poultry industry. For this reason, the AI techniques have been firstly described and well evolved in those bird species such as the domestic chicken [1], domestic turkey (*Meleagris gallopavo*) [2], and northern pintail (*Anas acuta*) [3], in which the AI technique has been integrated in the industrial process. The same technique has been adapted and transferred to other bird species and can be potentially used as a tool to increase the chances of survival for the endangered ones. There are a few reports of ex situ breeding conservation program that implements the use of an AI technique tool in association with in situ breeding program, which claims some successful results related to the use of this technique playing a consistent role in increasing numbers of certain species. The peregrine falcon is one of those species, with reports of more than 6000 captive-bred peregrine falcons released in nature among many states of the United States, putting this species on the top of the list of those that have benefited most from this conservation program. However, there are many flaws in expanding the use of AI techniques in endangered bird species, among which the lack of knowledge of basic and applied biology of reproduction, husbandry, chick rearing, and medical management in other nondomestic species [4]. Moreover, developing successful AI for raptors requires both reliable semen collection and a thorough evaluation of sperm quality characteristics. A recent study focusing on the variable morphological sperm characteristics of the peregrine falcon showed an increase of sperm head defects depending on the stage of the reproductive season, with an increase toward the end of season, and a certain degree of immature sperm throughout all the season [5].

Lastly, a semen extender has a pivotal role in the advancement of AI in breeding programs for preservation of threatened raptor species and of falconry for commercial purposes. Indeed, its use involves the possibility to delay AI by storing the collected semen for a limited amount of time after semen collection. Once diluted, raptor semen can be chilled and frozen and this could be a future interesting tool to be used in captive breeding programs, when specific identified individuals may be incompatible, for example, because of stress, thus leading to an absence of mating [6]. However, the effective use of AI depends on the extender's ability to provide an adequate environment for spermatozoa during storage, without altering sperm characteristics and function. Many extenders have been described for semen dilution in different avian species, with Beltsville Poultry Semen Extender (BPSE) or Lake's diluent most often used [7]. There is proof of cryotolerance of peregrine falcon and imperial eagle spermatozoa by adding dimethylacetamide (DMA) to semen extenders, and recently, a trial analyzed different protocols of freezing–thawing using two cryoprotectants (DMA and DMSO) in order to optimize the freezing protocol [8].

Tris-based extenders (TbEs) are some of the most popular extenders used in many species. They help in the AI performance with many effects, such as to dilute the semen sample at a suitable concentration and final volume, to give an energetic substrate, to be a pH buffer, and to control bacterial growth, and they are also used with the addition of a cryoprotectant for semen freezing extenders. However, to the best of the authors' knowledge, the use of TbEs in birds is poorly documented, as only a couple of studies reported Tris as a basic component for extender for turkey frozen spermatozoa [4, 9] and for short-term storage of rooster spermatozoa. The aim of this report was to gather information on the use of a TbE in maintaining motility and viability during short-time semen storage at room temperature in spermatozoa of a Gyr-saker hybrid falcon.

## 2. Case Presentation

### 2.1. Patient and Sample Collection

A hand-reared imprinted 11-year-old hybrid falcon (*Falco rusticolus* × *Falco cherrug*) was used as semen donor in order to perform AI procedures during the reproductive season. It was housed individually in an open-air facility under natural photoperiod in a private collection and breeding center (*Urbeveteris Falconis*, Orvieto, Italy); the handling of the raptor and semen collection were performed in accordance with the European and Italian legislation on species conservation [10, 11]. The donor was healthy, sexually mature, and of proven fertility at the beginning of this study.

A total of 15 successful semen collection procedures (out of 17) was carried out across 2 months (April–May) of two consecutive reproductive seasons. This proportion was defined as efficacy of sperm collection. For semen collection, the donor was gently restrained using a towel wrapped around the body and ejaculation was achieved by abdominal massage technique, with thumb and index moving from the cranial aspect of the abdomen toward the cloaca, followed by a gentle pressure at the base of the vent [12, 13]. This procedure was performed once daily, from two to four times a week during the reproductive season. Sperm samples were collected in 75-*μ*L nonheparinized and graduated microcapillary tubes (Vitrex Medical S/A, Herlev, Denmark). Ejaculates macroscopically contaminated with feces and urates were discarded from the study.

### 2.2. Sample Processing and Laboratory Testing

Color, volume, pH, concentration, viability, and motility were evaluated immediately after collection. A color score (0–3) was used for assessment considering both color and clearness (0, whitish-turbid; 1, whitish-clear; 2, pale yellowish-clear; 3, yellowish-turbid), while pH was qualitatively recorded using a universal indicator paper (VELP Scientifica, Usmate Velate, Italy). Sperm volume was determined by graduated microcapillary tube, measuring it directly during the collection. For sperm concentration, 5 *μ*L of diluted sample (1:40, semen:distilled water, *v*/*v*) were inserted into a Vetriplast counting chamber (Vacutest Kima, Arzergrande, Italy). This tool was chosen because it contains 10 different counting chambers on the same glass slide, and it allows the use of smaller quantities of seminal material compared to other conventional counting chambers.

Each sample was split in two aliquots, one was diluted (1:1, *v*/*v*) with a TbE having the following composition: Tris 3.025 g, citric acid 1.7 g, fructose 1.25 g, streptomycin 0.1 g, benzylpenicillin 0.06 g, and distilled water up to 100 mL (pH 6.76, 324 mOsm) [14]. The other one was left undiluted. Sperm motility was evaluated subjectively by a trained veterinarian on both undiluted and TbE-diluted semen samples at room temperature immediately (T0) and after 20 (T20) and 40 (T40) minutes from semen collection. Progressive motility intensity (PMI) score was evaluated on a 5-point scale (from 0, *no motility*, to 5, *very fast progressive motility*) [15].

Five microliters of undiluted or TbE-diluted sample was mounted on a prewarmed glass slide under a cover slip (18 × 18 mm) and evaluated on five microscopic fields (200×). Digital frames were acquired by a Nikon D3200 camera and with a NIS-Elements (v.5.30.00) viewer software (Nikon Corporation, Minato-Ku, Japan). Such videos were then used to categorize the percentage of sperm total motility (TM%), progressive motility (PM%), and nonprogressive motility (NPM%).

For sperm viability, 5 *μ*L of sample was transferred into 0.5-mL Eppendorf tubes and incubated for 30 s with 6.5 *μ*L of eosin/nigrosin dye (eosin G 2% and nigrosin 4%, Minitüb GmbH, Tiefenbach, Germany). Then, 5 *μ*L of stained sample was used for smear preparation. This procedure was repeated for both undiluted and TbE-diluted samples. Viability assessment was performed evaluating 200 spermatozoa for each smear, under 400× optical Olympus BX40 microscope (Olympus Corporation, Tokyo, Japan), and calculating the percentage of viable spermatic cells [16, 17].

### 2.3. Statistical Analyses

Descriptive normally distributed data (volume, pH, and sperm concentration) were reported as mean ± standard deviation (SD), whereas not normally distributed ones (color score) were reported as median (min–max). Comparison between undiluted and TbE-diluted semen for viability was performed using a nonparametric approach (Mann–Whitney *U* test). An ANOVA test was used to compare TM%, PM%, and NPM% considering the dilution effect and the three time intervals (T0, T20, and T40), as well as their interaction. Results of the ANOVA were reported as least square means (LSmeans) ± standard error (SE). Pearson's correlation was applied to test the correlation among PM% and PMI score, and Spearman's correlation was used to verify the correlation between sperm viability and PMI score. All statistical procedures were carried out using the release 9.4 of the SAS software (SAS Institute Inc., Cary, United States).

### 2.4. Results

The descriptive data of the efficacy of the semen collection and sperm characteristics in the hybrid falcon are shown in Table 1. Only one sample was discarded due to fecal and urate contamination and then removed from the study. No statistical differences on TM%, PM%, and NPM% were observed across the different time intervals in either the undiluted or TbE-diluted semen (Table 2). The use of the extender also resulted in similar sperm motility data, except for a slightly lower PM% values in undiluted samples (Table 2).

The median (min–max) PMI score observed for undiluted and TbE-diluted semen was 2.5 (2.0–4.0) and 3.0 (2.5–4.0), respectively. Spearman's correlation coefficients confirmed a highly significant and positive correlation between PM% and PMI score (*r* = 0.71, *p* = 0.009) only for undiluted semen. A significant correlation between sperm viability and PMI score on both undiluted (*r* = 0.67, *p* = 0.015) and TbE-diluted (*r* = 0.64, *p* = 0.028) samples was also observed. The percentage of viable spermatozoa, expressed as median (min–max), was 93% (83%–95%) for undiluted and 91% (75%–93%) for TbE-diluted semen samples. No significant (*p* = 0.486) difference in sperm viability was found between undiluted and TbE-diluted semen.

## 3. Discussion

Methods for collecting semen in raptors, described in the literature, include the cooperative method and the abdominal massage [12, 18]. The efficacy rate of sperm collection with abdominal massage can vary depending on the species and on the individual. In the present study, the efficacy rate (%) was 88.2 ± 14.9, an experimental outcome slightly greater compared to those reported with similar operative approach in other raptors like American kestrel (74%) [19] and Indian white-backed vulture (85%) [20]. Although it has been reported that the abdominal massage can lead to a higher percentage of feces and urates contamination, as it has been found in the golden eagle (37%), peregrine falcon (48%), and Indian white-backed vulture (39%) [20–21].

The mean volume of semen collected (84 ± 49* μ*L) was comparable with values reported for the parental species: 40 and 25 *μ*L for the Gyrfalcon [12, 22] and 45 *μ*L for the saker falcon [18]. These deviations are likely due to the inter- and intraindividual variability of semen donors and to the ability of the experienced operator in the semen collection operativity. Additionally, other studies have also reported that semen volume varies throughout the breeding season [23].

At time of collection (T0), the TM% on undiluted semen (32% ± 7%) is lower than what was estimated for the Gyrfalcon (68.8%) and the saker falcon (74.2%) [18]. Consequently, PM% (19% ± 5%) is lower compared to value reported in another study on the Gyrfalcon (75% ± 12%) [22]. These differences on sperm motility compared to other falcon species could be due to the housing condition of the patient. The age of the Gyrfalcon may also play a role. In a recent study, it was postulated that the best results in semen quality were obtained during the full reproductive season (i.e., April) and also that the quality of semen collected at the beginning of the season may be reduced with the increase of the age of the male [24]. In that study, the age interval of the birds of prey involved was in a range 5–7 years, while in our work the Gyrfalcon involved was 11 years. This advanced age of the hybrid falcon might be the cause of the lower sperm motility of the ejaculates; even if the motility reported in this work is not optimal, it is still worth to be considered as the animal was in any case used in the breeding facility for the AI procedures.

Even in literature, the motility reported for raptor species can vary widely, depending mostly on the moment of the reproductive season (e.g., best around April and May in south of Europe) and on the method of collection. In a previous work, a total motility of 18%–59.2% and a progressive motility of 3%–36.8%, in a total of 17 samples, were found in four different species of wild raptors (*Accipiter nisus*, *Falco subbuteo*, *Falco tinnunculus*, and *Bubo bubo*) using a CASA system [13]. Also, the method of semen analysis can play a role in the final results. Many laboratories not equipped with advanced computerized analysis system rely on more conventional methods such as the subjective evaluation of the motility samples. Given that subjective evaluation is valid whenever performed by trained laboratory professionals, it may however lead to subjective interpretation of sperm motility. In order to avoid bias due to subjective interpretation, usually the motility can be evaluated by two different operators independently, but in this work, this was not done. Despite the low value in motility percentages, the diluted and undiluted samples were then maintained at the same laboratory conditions, in order to avoid any other bias on the final results of motility, except for the time from collection and the dilution. Moreover, the estimated PMI score was confirmed by the positive and significant correlation with PM% data. In addition, sperm viability seems to be positively correlated with PM%. This confirmed that the operator was able to distinguish viable cells stained with eosin/nigrosin dye and that the vast majority of viable spermatozoa were characterized by progressive movement.

The dilution with TbE did not cause any significant difference in TM% and PM% with respect to undiluted samples in the three considered time intervals, suggesting that TbE seems to maintain a good sperm motility in samples stored at room temperature for at least 40 min. Furthermore, no significant difference was found in terms of viability between undiluted and diluted samples. This suggests that TbE did not cause alteration of the sperm membrane integrity. TbEs are often used as medium for long-term and short-term sperm storage in many species because of their buffer activity and because many other components like sugars and cryoprotectants can be added to maintain viability of spermatozoa at different conditions of temperature. In avian species, its use has never been systematically pointed out, and, to the authors' knowledge, only one recently published work used TbE as base to demonstrate the efficacy of the rosemary officinalis in the preservation of rooster semen at +4°C [25].

Although these results are obtained with semen repetitively sampled by just one subject, we considered them quite encouraging because no detrimental variation of semen quality for 40 min after collection at room temperature was found after semen dilution with a TbE. Further studies are needed and more robust results may be obtained whether the extender is tested in different conditions of temperature, for a longer time, and with a higher number of subjects enrolled, by comparing an adequate number of treated versus nontreated samples.

## 4. Conclusion

In this case report, it was possible to describe the absence of detrimental effects of a TbE in the motility parameters (TM% and PM%) by comparing the diluted and undiluted semen samples collected from a Gyr-saker hybrid falcon. Furthermore, no significant difference in the viability on undiluted and TbE-diluted semen was detected. TbE was not detrimental to the most basic sperm functions observed in this report, and the extender used was able to support sperm motility for approximately 1 h at room temperature. Further studies are needed to evaluate the efficacy of the semen dilution on a TbE by evaluating the motility and viability parameters for a longer time after dilution and in a larger number of animals, as well as before and after results of the AI procedure in multiple females during the reproductive season.

## Figures and Tables

**Table 1:** Descriptive statistics as mean ± standard deviation (SD) of the sperm parameters in Gyr-saker hybrid falcon.

**Parameter**	**Mean (±SD)**
Efficacy of sperm collection (%)^a^	88.2 ± 14.9
Volume (*μ*L)	84 ± 49
Color score (0–3)^b^	2 (0–2)
pH^b^	7.5 (7.0–7.6)
Concentration (spermatozoa ×10^3^/*μ*L)	109.2 ± 74.3

^a^Proportion of successful semen collection out of total.

^b^Color score and pH are reported as median (min–max).

**Table 2:** Effects of the extender on sperm total (TM%), progressive (PM%), and nonprogressive (NPM%) motility at different time intervals in Gyr-saker hybrid falcon.

**Parameter**	**Undiluted**				**TbE-diluted**				**p** **extender**
	**T0**	**T20**	**T40**	**p** **time**	**T0**	**T20**	**T40**	**p** **time**	
TM%	32 ± 7	36 ± 7	42 ± 7	0.552	43 ± 7	43 ± 7	45 ± 7	0.980	0.237
PM%	19 ± 5	21 ± 5	25 ± 5	0.620	29 ± 5	25 ± 5	32 ± 5	0.598	0.067
NPM%	14 ± 3	15 ± 3	19 ± 3	0.387	14 ± 3	20 ± 3	15 ± 3	0.401	0.904

*Note:* T0, T20, and T40 min from semen collection, respectively. Data are reported as LSmeans ± standard error. The interaction between extender and time intervals was never significant.

## Data Availability

The data that support the findings of this study are available from the corresponding author upon reasonable request.
